# A Comparative Study of Video-Assisted Thoracic Surgery with Thoracotomy for Middle Lobe Syndrome

**DOI:** 10.1007/s00268-016-3777-6

**Published:** 2016-11-02

**Authors:** Jian Li, Chengwu Liu, Yongsheng Zhao, Chuan Li, Lunxu Liu

**Affiliations:** 1 Department of Thoracic Surgery, West China Hospital, Sichuan University, No. 37, Guoxue Alley, Chengdu, Sichuan 610041 China; 2 Western China Collaborative Innovation Center for Early Diagnosis and Multidisciplinary Therapy of Lung Cancer, Sichuan University, Chengdu, 610041 China

## Abstract

**Objectives:**

The aim of this study is to evaluate the feasibility and safety of video-assisted thoracic surgery (VATS) for the treatment of middle lobe syndrome (MLS) through comparison with thoracotomy during the same period.

**Methods:**

We retrospectively reviewed all consecutive patients with MLS who underwent lobectomy or lingular segmentectomy between December 2005 and November 2015 in a single institute. Thirty patients were enrolled and divided into two groups: VATS group (*n* = 19) and thoracotomy group (*n* = 11). Data regarding the patients’ demographics, medical history were collected and statistically compared.

**Results:**

All patients received successful middle lobe resection or lingular segmentectomy. In terms of operation time, blood transfusion, chest drainage amount, duration of chest drainage and postoperative complications, no significant differences were found between the two groups (*p* > 0.05). The mean intraoperative blood loss of VATS group was less than thoracotomy group (79.0 ± 63.9 vs. 165 ± 94.9 ml, *p* = 0.04). In VATS group, the mean length of postoperative hospital stay was 6.0 ± 2.4 days, shorter than that in group thoracotomy (9.0 ± 3.5 days, *p* = 0.01).

**Conclusions:**

VATS was a feasible and safe method for the surgical treatment of MLS in selected patients when no severe calcified lymph nodes surrounding hilus pulmonis was observed by preoperative chest CT scan.

## Introduction

The name of “middle lobe syndrome” (MLS) was suggested by Graham et al. [1]. Extraluminal or intraluminal bronchial obstruction, recurrent atelectasis of the right middle lobe or left lingular segment was defined as MLS [2]. Most patients with MLS would recover by applying antibiotics and bronchodilators, especially for non-obstructive type. Surgical intervention would be necessary for approximately one-third patients with MLS if conservative therapy fails, or complete bronchial obstruction [3–6]. Lobectomy or lingular segmentectomy was an effective surgical treatment of MLS. In 1948, Graham and Burford reported 12 patients with MLS underwent middle lobe lobectomy and detailed the intraoperative findings. After that, Fretheim and other authors sporadically reported surgical treatment for MLS (see Table 1). Among these studies, the majority of surgical procedures were performed via thoracotomy [7–10].

**Table 1:** Surgical treatment and postoperative outcomes of patients with MLS

Authors	Number of patients and (operation)	Surgical procedure	Outcome
Graham et al. [1]	12 (12)	Middle lobe lobectomy for all patients	All were free of symptoms
Fretheim [7]	19 (9)	Middle lobe lobectomy for eight patients	Expectoration persisted in one patient after operation
Albo and Grimes [8]	99 (38)	Middle lobe lobectomy or right pneumonectomy for 38 patients	Completely symptom-free after right middle lobectomy
Saha et al. [4]	98 (31)	Middle lobe lobectomy for 29 patients Wedge resection for two patients	Recurrent symptoms presented in one patient Pleural effusion developed in three people, wound infection in one and atelectasis in one One patient died of pulmonary embolus postoperatively
Einarsson et al. [9]	18 (18)	Middle lobe lobectomy for all patients	One patient was reoperated for bleeding and air leakage. Thirteen patients were cured; five other patients were improved
Seitz et al. [13]	3 (3)	Thoracoscopic middle lobe resection for three patients	One patient underwent secondary procedures
Gudbjartsson and Gudmundsson [17]	20 (20)	Middle lobe resection for all patients	One patient died One patient underwent secondary procedures because of massive drainage amount
Pejhan et al. [6]	40 (40)	Middle lobe lobectomy for 37 patients Bronchotomy and tumor local resection for one patient Lower and middle bilobectomy for one patient Upper + middle sleeve lobectomy for one patient	All were cured

Patients except in Seitz’ study underwent thoracotomy surgery

Over the past decade, with the continuous development of VATS technique, VATS has been applicable for most thoracic pathologies [11]. However, only few authors [12–14] reported VATS for the treatment of MLS. In this paper, we’d like to report one of the largest case series of MLS treated via VATS compared with traditional thoracotomy.

## Materials and methods

This study included consecutive patients with MLS who were treated in the Department of Thoracic surgery of West China Hospital, Sichuan University between December 2005 and November 2015. According to surgical types, these patients were divided into VATS group (group V) and thoracotomy group (group T). Clinical data of each patient were collected and reviewed. The study protocol was approved by the institutional review board of West China Hospital.

All patients received preoperative chest computed tomography (CT) scan and fiberoptic bronchoscopy. All patients were placed in the lateral decubitus position under general anesthesia with double-lumen endotracheal tube and underwent middle lobe resection or lingular segmentectomy. With respect to surgical treatment, we have no special technique than others. Classic three-portal technique was used during VATS procedures. The observation port (1 cm), utility port (3 cm) and assistant incision (1.5 cm) were placed in the 7th intercostal space at the mid-axillary line, the 3rd intercostal space at the anterior axillary line and the 9th intercostal space between the posterior axillary line and subscapular line, respectively. Both of the VATS right middle lobectomy and left lingular segmentectomy were performed following the concept of single direction as we described before [15]. Thoracotomy was performed through a classic posterolateral incision (20–30 cm) made in the 4th intercostal space. Tissues were sent to frozen section examination routinely. One chest tube would be placed at the end of each operation. All procedures were performed by thoracic surgeons who have the similar experience. Chest tube was removed when the daily drainage was <200 mL and when no air leak was identified. Patients were discharged from the hospital when there was no main complication.

Data of each patient were collected from the medical records. Including gender, age, surgical approach, duration of operation, intraoperative blood loss, blood transfusion, chest drainage amount, duration of chest drainage, postoperative complications and the length of postoperative hospital stay.

Statistical Product and Service Solutions (SPSS) software versions 17.0 (SPSS, Inc, Chicago, IL, USA) was used to analyze data. The Chi-square test was used to analyze categorical variables, and Mann–Whitney Wilcoxon test was used to analyze continuous variables. *p* value <0.05 was considered statistically significant.

## Results

A total of 30 patients with MLS were enrolled, including 21 females and 9 males. The baseline demographic characteristics of the two groups are shown in Table 2. The mean age was 44.7 years (range 22–64 years). Among the 30 patients, 29 (96.7%) presented signs and symptoms prior to operation: cough with sputum production (group V ten cases; group T seven cases), cough with hemoptysis (group V five cases; group T three cases), chest pain (group V two cases; group T one cases) and dyspnea (group V one cases; group T 0 case). The other one patient was completely asymptomatic. Non-obstructive type accounts for 73.3% (group V 15 cases; group T 7 cases), the remaining 26.7% were obstructive type (group V four cases; group T four cases). The lesions were located in right middle lobes (group V 17 cases; group T 11 cases) or lingulars (group V two cases; group T 0 case). All patients received lobectomy or segmentectomy, 19 patients (63.3%) underwent VATS and 11 patients (36.7%) underwent thoracotomy.

**Table 2:** Demographic and baseline characteristics of all patients

Variables	Group V (*n* = 19)	Group T (*n* = 11)	*p* value
Gender			0.119
Male	4	5	
Female	15	6	
Age (years)	42.5 ± 11.3	40.9 ± 17.5	0.764
Main symptoms			
Cough + sputum production	10	7	
Cough + Hemoptysis	5	3	
Chest pain	2	1	
Dyspnea	1	0	
Others	1	0	
Category			0.433
Obstruction	4	4	
Non-obstruction	15	7	
Location of lesions			0.617
Right middle lobe	17	11	
Lingular	2	0	

*Group V* VATS group, *group T* thoracotomy group

Two cases in group V were converted to open procedure. The incidence was 10.5%. The reasons were dense adhesions and enlarged calcified lymph nodes located in hilus pulmonis, respectively. During the operation for the second case, the middle lobe pulmonary artery was injured when dissecting the middle lobe bronchus, with blood loss of approximately 700 mL. Anatomical lobectomy was performed for 24 patients (group V 15 cases; group T 9 cases). Anatomical lingular segmentectomy was performed for two patients (group V two cases; group T 0 case). Non-anatomical lobectomy was performed for four patients (group V two cases; group T two cases).

No significant differences were found between the two groups in the duration of operation, blood transfusion, chest drainage amount, duration of chest drainage and postoperative complications (*p* = 0.326, 0.716, 0.076, 0.346, 0.454, respectively, see Table 3). The mean intraoperative blood loss of group V was 79.0 ± 63.9 mL, less than that in group T (165.0 ± 94.9 mL, *p* = 0.04). The mean postoperative hospital stay length of group V was 6.0 days (range 3–15 days), shorter than in group T (9.0 days, range 4–17 days, *p* = 0.01); the differences were statistically significant. Only one patient of group T received blood transfusion. The most common pathology of MLS in the study was inflammation (*n* = 21), followed by bronchiectasis (*n* = 3), granulomatous (*n* = 1) and tuberculosis (*n* = 1). One patient received anti-tuberculosis treatment after confirmed tuberculosis by pathology. There was no intraoperative and postoperative mortality. Long-term postoperative follow-up was not analyzed.

**Table 3:** Perioperative clinical data of all patients

Variables	Group V (*n* = 19)	Group T (*n* = 11)	*p* value
Surgical method			
Anotomical lobectomy	15	9	
Anotomical segmentectomy	2	0	
Unanotomical lobectomy	2	2	
Duration of operation (min)	122.3 ± 33.4	150.0 ± 67.3	0.326
Intraoperative blood loss (ml)	79.0 ± 63.9	165 ± 94.9	0.04
Blood transfusion	0/19	1/11	0.716
Chest drainage amount (ml)	307.5 ± 231.0	510.4 ± 408.3	0.076
Duration of chest drainage (days)	2.9 ± 1.3	3.6 ± 1.8	0.346
Histology			
Infection	12	9	
Bronchiectasis	2	1	
Granulomatous	1	0	
Tuberculosis	1	0	
Others	3	1	
Complications	0	2	0.454
Pulmonary atelectasis	0	1	
Air leakage	0	1	
Postoperative hospital stay (days)	6.0 ± 2.4	9.0 ± 3.5	0.01

## Discussion

MLS is an uncommon clinical entity. Originally, Graham and his colleagues [1] defined this syndrome as atelectasis of middle lobe caused by enlarged lymph nodes compressing the middle lobe bronchus. During the past 60 years, the definition of MLS has been changed. Bronchial obstruction, recurrent atelectasis no matter located in the right middle lobe or lingular segment was defined as MLS [2]. However, MLS was predominantly located in right middle lobe. Except that the middle lobe bronchus has a narrow diameter and an angular takeoff from intermediate bronchus, there are two other factors: Firstly, the right middle lobe is relatively isolated compared with left lingular segment. Therefore, more space adjacent to bronchus was left for development and enlargement of neoplasm or lymph nodes. Secondly, as other authors reported [16], right middle lobe lacks collateral ventilation, and it was the other factor that right middle lobe was vulnerably to suffer MLS.

Although almost all patients with MLS presented syndromes preoperatively, the diagnosis of MLS is still hard only with medical history. Chest CT scan and fiberoptic bronchoscopy were necessary for diagnosis of MLS [4, 17]. The image of chest CT can show the location of lesions, surrounding the bronchus. Via fiberoptic bronchoscopy, intraluminal bronchus can be observed and the pathological diagnosis of MLS would be made. These two methods were useful for classification of MLS and preoperative evaluation. In this study, all patients received chest CT scan and fiberoptic bronchoscopy examination; no one was misdiagnosed.

According to the situation that the involved bronchus was completely obstructed or not, MLS can be classified into obstructive and non-obstructive types. Endobronchial tumour or tuberculosis, foreign bodies often cause the obstructive type [18, 19], while benign inflammation is the main cause of non-obstructive type. In this study, we found that operation for obstruction type was more difficult than that for non-obstruction type. When the lesion was located near the orifice of the bronchus, sleeve lobectomy was necessitated to make sure of negative stump especially when there was a neoplasm. For cases with outside compression caused by extraluminal tumor or lymph nodes, there might be no space between the lesion and the bronchus and dissection would be rather difficult.

The risk of conversion to thoracotomy was 2–23% in VATS lobectomies, and when the involved bronchus was surrounded by calcified lymph nodes, it would rise to 37% [20, 21]. In some cases, anatomical dissection of the vessels and bronchus could not be achieved because of dense adhesions between them. Sometimes clamping the pulmonary trunk would be helpful for the following safe dissection. However, for some cases, even if we used sharp dissection or converted to thoracotomy, dissection of the vessels and bronchus was still impossible. Then, we might transect the bronchus and pulmonary vessels together using endostapler. After the transection, a 3–0 prolene suture was used to reinforce the stump to prevent bronchopleural fistula and bleeding. In this study, there was no postoperative bleeding or bronchopleural fistula in the four patients who underwent non-anatomical lobectomy.

Several limitations of our analysis were realized as follows: (1) Our study is a retrospective review; it cannot reach random assignment to treatment. (2) Although all procedures were performed by surgeons with similar experience, differences would present inevitably. And this study lacked unified criteria to select patients for VATS or thoracotomy. (3) This study only analyzed short-term patient data; hence, the long-term outcomes of VATS for MLS cannot be assessed. Considering the different surgical skills and experience of the surgeons, as well as the small patients cohort, the results did not indicate that all patients with MLS would benefit from VATS. However, considering the unusual entity of MLS, this study at least provided one of the largest cohort of patients undergoing VATS procedures. More experience of VATS in the surgical treatment of MLS is expected. We suggest that VATS resection could be performed for selected patients when no severe calcified lymph nodes surrounding hilus pulmonis are observed by preoperative chest CT scan.

## Conclusions

In summary, our study indicated that VATS was a feasible and safe method for the surgical treatment of MLS in selected patients by experienced hands.

## References

[1] Graham EA, Burford TH, Mayer JH (1948). Middle lobe syndrome. Postgrad Med.

[2] Rosenbloom SA, Ravin CE, Putman CE (1983). Peripheral middle lobe syndrome. Radiology.

[3] Ayed AK (2004). Resection of the right middle lobe and lingular in children for middle lobe/lingual syndrome. Chest.

[4] Saha SP, Mayo P, Long GA (1982). Middle lobe syndrome: diagnosis and management. Ann Thorac Surg.

[5] Wagner RB, Johnston MR (1983). Middle lobe syndrome. Ann Thorac Surg.

[6] Pejhan S, Salehi F, Niusha S (2015). Ten years’ experience in surgical treatment of right middle lobe syndrome. Ann Thorac Cardiovasc Surg.

[7] Fretheim B (1952). The so-called middle lobe syndrome. Thorax.

[8] Albo RJ, Grimes OF (1966). The middle lobe syndrome: a clinical study. Dis Chest.

[9] Einarsson JT, Einarsson JG, Isaksson H (2009). Middle lobe syndrome: a nationwide study on clinicopathological features and surgical treatment. Clin Respir J.

[10] Sehitogullari A, Sayir F, Cobanoglu U (2012). Surgical treatment of right middle lobe syndrome in children. Ann Thorac Med.

[11] Caronia FP, Fiorelli A, Ruffini E (2014). A comparative analysis of Pancoast tumour resection performed via video-assisted thoracic surgery versus standard open approaches. Interact CardioVasc Thorac Surg.

[12] Rothenberg SS (2003). Experience with thoracoscopic lobectomy in infants and children. J Pediatr Surg.

[13] Seitz G, Warmann SW, Szavay PO (2010). Thoracoscopic lobectomy as a treatment option for persistent middle lobe syndrome in children. Pediatr Int.

[14] Yu JA, Pomerantz M, Bishop A (2011). Lady Windermere revisited: treatment with thoracoscopic lobectomy/segmentectomy for right middle lobe and lingular bronchiectasis associated with non-tuberculous mycobacterial disease. Eur J Cardiothorac Surg.

[15] Liu L, Che G, Pu Q (2010). A new concept of endoscopic lung cancer resection: single-direction thoracoscopic lobectomy. Surg Oncol.

[16] Inners CR, Terry PB, Traystman RJ (1978). Collateral ventilation and the middle lobe syndrome. Am Rev Respir Dis.

[17] Gudbjartsson T, Gudmundsson G (2012). Middle lobe syndrome: a review of clinicopathological features, diagnosis and treatment. Respiration.

[18] Gudmundsson G, Gross TJ (1996). Middle lobe syndrome. Am Fam Physician.

[19] Chien HP, Lin TP, Chen HL (2000). Right middle lobe atelectasis associated with endobronchial silicotic lesions. Arch Path Lab Med.

[20] McKenna RJ, Houck W, Fuller CB (2006). Video-assisted thoracic surgery lobectomy: experience with 1,100 cases. Ann Thorac Surg.

[21] Samson P, Guitron J, Reed MF (2013). Predictors of conversion to thoracotomy for video-assisted thoracoscopic lobectomy: a retrospective analysis and the influence of computed tomography-based calcification assessment. J Thorac Cardiovasc Surg.

